# Effect of Sago Starch Modifications on Polystyrene/Thermoplastic Starch Blends

**DOI:** 10.3390/ma14112867

**Published:** 2021-05-27

**Authors:** Mohamad Kahar Ab Wahab, Halimatul Syahirah Mohamad, Elammaran Jayamani, Hanafi Ismail, Izabela Wnuk, Anna Przybył, Tomasz Stachowiak, Przemysław Postawa

**Affiliations:** 1 Faculty of Chemical Engineering Technology, Universiti Malaysia Perlis (UniMAP), Kangar 06100, Perlis, Malaysia; hallyra93@gmail.com; 2 Geopolymer & Green Technology, Centre of Excellence (CEGeoGTech), Universiti Malaysia Perlis (UniMAP), Kangar 06100, Perlis, Malaysia; 3 Faculty of Engineering, Computing and Sciences, Swinburne University of Technology Sarawak Campus, Kuching 93350, Sarawak, Malaysia; ejayamani@swinburne.edu.my; 4 School of Materials and Mineral Resources Engineering, Engineering Campus, Universiti Sains Malaysia, Seri Ampangan Nibong Tebal, Seberang Perai Selatan, Nibong Tebal 14300, Penang, Malaysia; ihanafi@usm.my; 5 Department of Physics, Częstochowa University of Technology, 42-216 Częstochowa, Poland; wnuk.izabela@wip.pcz.pl (I.W.); przybyl.anna@wip.pcz.pl (A.P.); 6 Faculty of Mechanical Engineering and Computer Science, Częstochowa University of Technology, 42-216 Częstochowa, Poland; sachowiak@ipp.pcz.pl (T.S.); postawa@ipp.pcz.pl (P.P.)

**Keywords:** thermoplastic starch, sago starch, chemical modification

## Abstract

The preparation of polystyrene/thermoplastic starch (PS/TPS) blends was divided into three stages. The first stage involved the preparation of TPS from sago starch. Then, for the second stage, PS was blended with TPS to produce a TPS/PS blend. The ratios of the TPS/PS blend were 20:80, 40:60, 60:40, and 80:20. The final stage was a modification of the composition of TPS/PS blends with succinic anhydride and ascorbic acid treatment. Both untreated and treated blends were characterized by their physical, thermal, and surface morphology properties. The obtained results indicate that modified blends have better tensile strength as the adhesion between TPS and PS was improved. This can be observed from SEM micrographs, as modified blends with succinic anhydride and ascorbic acid had smaller TPS dispersion in PS/TPS blends. The micrograph showed that there was no agglomeration and void formation in the TPS/PS blending process. Furthermore, modified blends show better thermal stability, as proved by thermogravimetric analysis. Water uptake into the TPS/PS blends also decreased after the modifications, and the structural analysis showed the formation of a new peak after the modification process.

## 1. Introduction

Over the years, there has been various research and developments in producing blends by incorporating natural polymers into synthetic polymers. In principle, the biodegradability of synthetic polymers can be improved by blending natural polymers. Polymer blending is a popular method whenever modification of properties is required because it uses customary technology at a low cost. The common goal for preparing a novel blend of two or more polymers is not to change the properties of the constituents radically but to capitalize on the maximum possible performance of the blends [1,2,3]. The major drawback of conventional plastics is low biodegradability when they were disposed of in typical solid waste landfills. They are called non-biodegradable polymers as they are not decomposed by microorganisms, and their carbon component cannot be broken down by the enzymes of microorganisms [4]. Increasing the waste material in landfills contributes to significant problems such as pollution of groundwater and surface water, which affects humans and other living organisms. According to Santos et al., conventional plastic causes worse consequences for the environment due to common polyolefin, which has a very low degradation rate [5]. These facts were supported by Fischer et al., who stated that a higher cost is incurred for storing non-biodegradable plastic. Hence, the option of burning or incineration becomes economically viable. Although burning these plastics emits carbon dioxide (CO_2_), which contributes to green house effects resulting in global warming [6].

To address this problem, many studies have been carried out to develop biodegradable plastic that can be applied, especially in food packaging and agriculture [7]. Biodegradable plastics are derived from renewable natural resources and have become a viable alternative in order to decrease the pollution problems [8]. Biodegradable plastics are polymers that can be decomposed via microbial activities such as bacteria, algae, and fungi. Examples of naturally derived biodegradable plastics are materials made from starch, chitosan, and lignocellulosic base [9,10]. Starch became a major choice for producing biodegradable plastics due to its renewable aspects, abundant availability, and low cost. However, starch itself does not exhibit thermoplastic behavior due to intramolecular and intermolecular hydrogen bonds between hydroxyl groups of starch structures, which stands for their crystallinity exits in a granular form [11]. Various methods have been developed to improve the flow characteristics of starch, such as the incorporation of a plasticizer, namely glycerol [12]. The addition of plasticizers leads to disruption of the semi-crystalline structure and results in an amorphous material called thermoplastic starch (TPS). However, thermoplastic starch possesses its own drawbacks, such as low mechanical properties, highly brittle characteristics, and low water resistance. These limitations are attributed to the hydroxyl group present on the starch molecule that makes it hydrophilic in nature and has limited performance. Hence, blending with other synthetic polymers such as polystyrene is one of the approaches to improve its properties [13,14].

Furthermore, succinic anhydride and ascorbic acid were chosen for surface treatments in order to improve blend compatibility. TPS moisture sensitivity can be reduced by altering the hydroxyl groups of TPS with the hydrophobic groups from succinic anhydride. Thermoplastic starch was treated with succinic anhydride to increase the hydrophobicity of native starch by disruption of hydrogen bonding. Previous studies show that the estification process could be obtained via modification of TPS with succinic anhydride [15,16]. The success of TPS esterification depends on some factors such as reactant concentration pH and reaction of time [17]. According to Bhosale and Singhal, native starch which is modified by treating with octanoyl succinic anhydride (OSA), which includes the incorporation of alkenyl groups and gives the surface-active properties to the starch [18]. From the previous study, Bengtsson et al. reported that the surface energy of high amylose potato starch films was lower and decreased in water absorption after surface esterification with octanoyl chloride and pyridine [19]. On the other hand, the addition of ascorbic acid in TPS might act as a crosslinking agent and produce the network within the starch chains and enhance the dimensional stability of TPS. The weak hydrogen bonding between starch chains is replaced by a stronger crosslinked bond. Consequently, solubility of the crosslinked TPS in water is reduced and results in higher freeze thaw stability, and a higher acid, shear, and heat stability [20,21,22,23]. Singh et al. also stated that the crosslinked starch is more resistance against acidity and harsh shear compared to the native starch [24].

In the present study, sago starch was used as a viable alternative for conventional plastics and was blended with polystyrene in the presence of glycerol as a plasticizer. The aim of the modified TPS with succinic anhydride and ascorbic acid were to obtain novel thermoplastic starch with the targeted properties that may benefit processability, good dimension stability, and improve the compatibility when blended with polystyrene.

## 2. Materials and Methods

Polystyrene with a density of 0.92 g/cm^3^ was supplied by Lotte Chemical Titan (Johor, Malaysia). Food grade sago starch was used to prepare thermoplastic starch in the presence of glycerol as the plasticizer. The granular sizes of sago starch ranged from 10 to 35 μm. Sago starch was used due to its unique properties of having a smooth granule surface with some pitting and large granular size. Glycerol with 99% purity was supplied by TTD Chemical (Johor, Malaysia). Reagent grade ascorbic acid, and succinic anhydride was purchased from Sigma Aldrich (St. Louis, MI, USA).

### 2.1. Sago Starch Modification Succinic Anhydride

A total of 300 g of sago starch was suspended in 700 mL of distilled water and continuously stirred. The starch solution was adjusted to a pH of 8.5 (alkali solution) in 3 vol% of NaOH solution. Next, a solution of 30 g succinic anhydride in 70 mL of distilled water was added to the starch slurry for 2 h. The pH range of the system was maintained at a given level with 3% NaOH solution until the reaction was completed. After the reaction, the pH was adjusted to 6.5–7.0 using 5% hydrochloric acid (HCl) solution. The mixture was then sieved and washed with ethanol and distilled water, and the solid was dried at 50 °C for 12 h.

### 2.2. Preparation of Thermoplastic Starch (TPS)

TPS was prepared in the ratio of 65:35 starch to glycerol according to our previous study [14]. After mixing, the paste was left for 24 h at room temperature. Later, TPS was prepared by using Gotech heated two roll mills at a temperature of 120 °C for 10 min. A similar method was applied to prepare 3% ascorbic acid-treated thermoplastic starch as well.

### 2.3. Preparation of TPS/PS Blends

The PS was added into the heated two roll mill until all the PS pellets were molten. TPS was then added to blend together with the molten PS until a homogeneous appearance of the plastic mixture was observed. The mixing process was conducted using two heated roll mills with a speed of 15 rpm at 200 °C for 15 min. The blends were then molded into 1 mm sheets through a Gotech compression molding machine at 200 °C for mechanical testing and characterization purposes. Table 1 shows the composition of TPS blended with PS in varying weight percentages.

### 2.4. Characterization and Mechanical Testing

Measurements of the tensile properties were performed by using an Instron Universal Testing Machine (model 3366, Instron, Norwood, MA, USA), with a crosshead speed of 50 mm/min. The tensile test was used to investigate tensile strength, tensile modulus, and elongation at break of the blends with the average value of 5 specimens for each compound. Fourier transform infrared spectroscopy (FTIR) of the blends were measured using a Perkin Elmer System 2000 (Perkin Elmer, Massachusetts, United States) to characterize the possible reaction between TPS, succinic anhydride, and ascorbic acids. The transmittance spectra regions were obtained between 4000 cm^−1^ and 800 cm^−1^. The fracture surface of the samples was observed by a Leo Supra−3SVP field emission scanning electron microscopy (SEM, Zeiss, Oberkochen Germany) at an acceleration voltage of 20 kV. The fracture surfaces of the specimen were mounted on aluminum stubs and sputter-coated with a thin layer of gold to avoid electrostatic charge during the examination. The thermogravimetric analysis (TGA) of the blends was operated using a Perkin Elmer Pyris 6 machine (Perkin Elmer, Massachusetts, United States). The thermal stability of the TPS/PS blends was tested at a heating rate of 20 °C/min from ambient temperature to 600 °C under a nitrogenous environment. For testing water absorption, samples with dimensions of 11 mm × 11 mm and 2.5 mm thick were fabricated and were dried for 24 h at 105 °C. The amount of water absorbed by the sample was determined by weighing them periodically until a constant value was obtained.

## 3. Results

### 3.1. Tensile Properties of TPS/PS Blends

**Figure 1:**
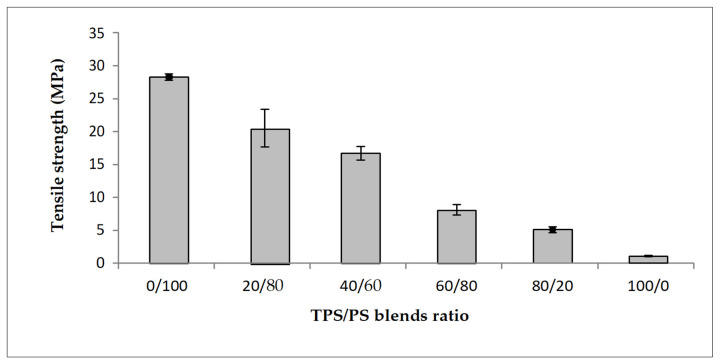
Tensile strength of untreated TPS/PS blends at different ratios.

**Figure 2:**
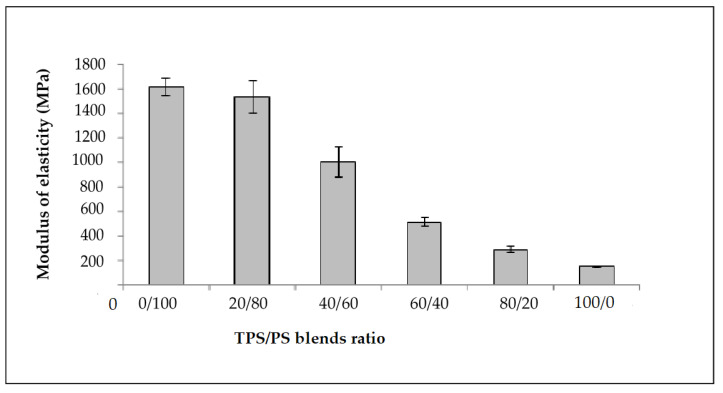
Modulus of elasticity of untreated of TPS/PS blends at different blends ratio.

**Figure 3:**
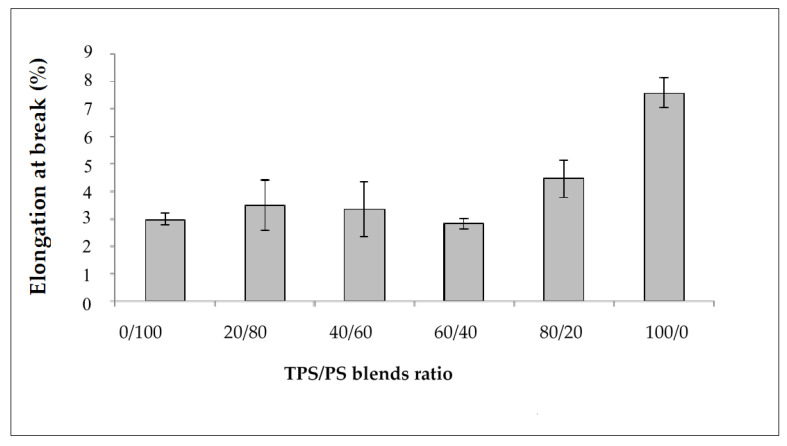
Elongation at break of TPS/PS blends at different ratios.

The effect of different blends proportion on tensile strength of TPS/PS blends is shown in Figure 1. The results show that the highest content of polystyrene in the blends produces the highest tensile strength. The higher content of polystyrene in the blends gives better results due to the toughness of PS material in the blends. The tensile strength value showed a continuous decrease with increasing TPS content. TPS acts as an inert compound and increasing TPS in the blends leads to the reduction of tensile strength [25]. At higher TPS loading, large phase formation of TPS occurs, which is due to the lack of dispersion in the PS matrix. These large TPS phases increase the stress–concentration points in the blend, which resulted in the tensile strength failure at a lower strain [26]. Figure 2 shows the modulus of elasticity TPS/PS at different blends. Different blend ratios show that the neat PS had the highest modulus compared to other ratios. The modulus gradually decreased when adding the TPS. The modulus decreased due to the effect of incorporation of soft materials in TPS/PS blends. Adding more TPS could also interfere with the PS chain alignment. The presence of glycerol on TPS loading also affects the modulus of elasticity of the blends as it makes the blends become softer and reduces the stress transfer between the blends. Taquet et al. [27] found that the blend with a higher amount of glycerol was over plasticized, and the glycerol softens the TPS/PS blends. Similar results were reported by Godbole and co-workers, which found that adding starch into a thermoplastic system decreased the modulus [28]. Figure 3 shows the effect of elongation at break on the TPS/PS blending with different blend proportions. At 0 and 20 wt% of TPS content, there was a slight increase in elongation at break. Further increasing the TPS content increases elongation at break except for 60 wt% of TPS loading. However, at 100 wt% TPS loading, the elongation break shows the highest value as compared to other ratios. This observation is due to neat TPS softening the blends, and that allows for better ductility, resulting in an increase of elongation. There was a slight decrease in elongation at break with 60 wt% of TPS loading, which might be due to weak dispersion of PS particles in the TPS component and thus initiate breaking when the force is applied.

### 3.2. Tensile Properties of Treated TPS/PS Blends

**Figure 4:**
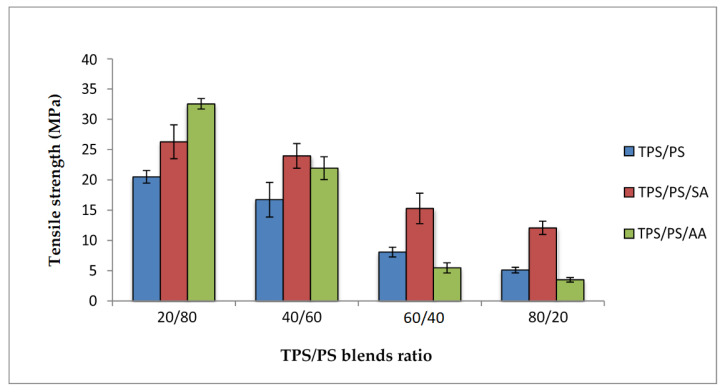
Tensile strength of treated TPS/PS with AA and SA at different blend ratios.

Figure 4 shows the tensile strength of untreated and treated TPS with succinic anhydride (SA) and ascorbic acid (AA) at different blend proportions of TPS/PS. All the blends indicated that higher TPS loading decreased the tensile strength. This was due to the poor interfacial adhesion and ability of TPS to support stress transfer from the PS component. Immiscibility of the TPS and PS component reduces the dispersion of blends. Treated TPS/PS blends with SA exhibit higher strength for all proportions as compared to untreated blends. This modification decreased the hydrophilic sensitivity of the blends by the esterification process. Thus, it can be understood that this treatment had improved the surface adhesion between the blends and increased compatibility at the interphase of TPS blends incorporating the starch surface. Similar results were also reported by Shogren et al. [29], who reported that esterification is one of the modifications which can impart hydrophobicity to starch products. Untreated TPS/PS blends gave lower strength due to incompatibility between the hydrophilic TPS and hydrophobic PS that causes weak interaction between the blends. It has also been reported that lowering starch molecular weight via the hydrolysis process in diluted acid can improve adhesion at the interphase and thus improved the compatibility between TPS-PS components. However, the tensile strength of the blends treated with ascorbic acid reduces when the TPS loading was increased. The primary reason for this reduction is that ascorbic acid promotes the acidolysis of TPS, resulting in a reduction of the tensile strength of the blends. At 60/40 and 80/20 TPS/PS blends, the TPS phase becomes a major component and affects the overall performance of the blends. It is due to the TPS, in the presence of a plasticizer, which acts as a softening material and is unable to support the external stress applied to the blends. The graphs show the gradual decrease in tensile strength for all the blends. However, the effect of plasticized TPS becomes more prominent for untreated and ascorbis acid modified TPS. Although the treatment with ascorbic acid can improve the plasticization process, the acidity of ascorbic acid also causes partial hydrolysis on the starch polymer chains. This will result in severe reduction in tensile strength especially for the higher TPS loading system.

**Figure 5:**
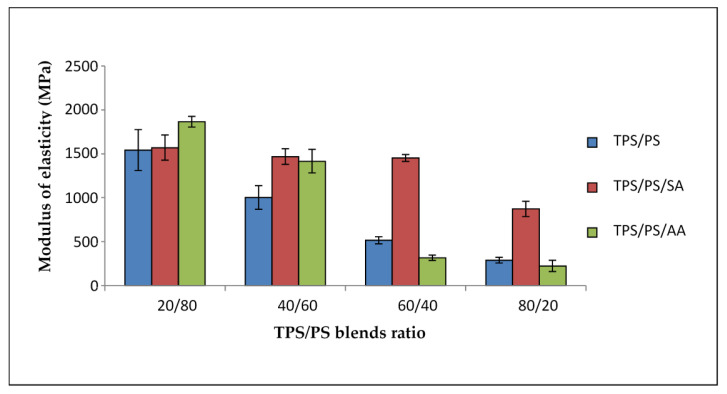
Modulus of elasticity of treated TPS/PS with AA and SA at different blend ratios.

Modulus elasticity of untreated and treated blends is illustrated in Figure 5 with different proportions of blends. Three significant trends in the figure showed that the modulus of the blends decreased when the TPS loading in the blends was increased. For untreated TPS/PS blends, these trends of the graph show a continuous reduction at higher TPS loading. Inference can be drawn in this observation that TPS reduced the stress transfer on the PS surface, thus reducing the modulus of elasticity. Treated TPS/PS blends with SA showed a similar graph for 20 wt% until 60 wt% of TPS loading. At 80 wt% of TPS loading, the modulus of TPS/PS/SA slightly decreases due to a higher number of the hydroxyl groups on TPS that make the interactions weak. Treated blends with ascorbic acid showed a gradual decrease for 20 wt% and 40 wt% of TPS loading. However, higher TPS loading at 60 wt% and 80 wt% showed a major drop of modulus value. This is because, at lower TPS loading, partial hydrolysis of starch chains increased the adhesion of the surface. However, when TPS acts as a major component, the effect of hydrolysis of TPS becomes prominent, and the degradation of TPS will affect the overall performance of the blend system. Treated TPS/PS with SA offered better modulus of elasticity as the treatment only occurs at surface modification, which imparts the hydrophobicity compared to blends treated with AA. Blends treated with AA will cause partial hydrolysis, which will affect the modulus result when TPS content is higher.

**Figure 6:**
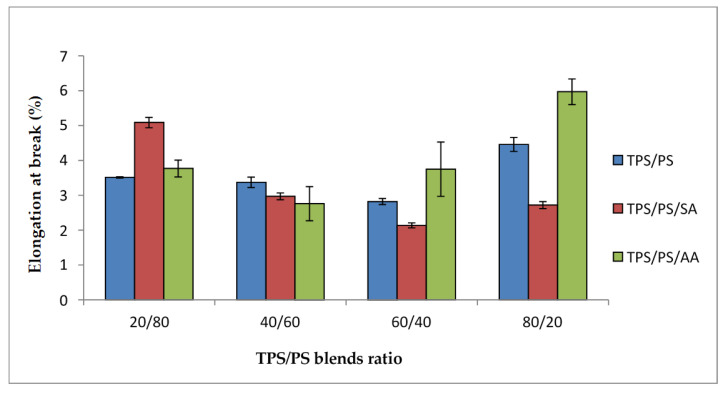
Elongation at break tensile of treated TPS/PS with AA and SA at different blend ratios.

Figure 6 shows the elongation at break of TPS/PS with different blend proportions. Generally, all the blend systems show that the value tends to decrease at higher TPS loading. The interaction between TPS-PS at the interphase had prevented TPS from elongating further due to the brittle character of the PS component. However, a different trend was observed for the TPS/PS/AA blends system. The elongation at break for TPS/PS/AA blends shows a significant increase, especially when TPS content was higher in the blends. This resulted from the partial hydrolysis that occurred in the TPS phase, which reduces the blends’ viscosity and thus increases the ability of the blends to elongate further. According to the study carried out by Mohd Zain et al. [30], incorporation of ascorbic acid gives better processability and improves the ductility of TPS in the blends. The effect of partial hydrolysis in the TPS phase was clearly observed for the TPS/PS modified with ascorbic acid especially at higher TPS loading. This phenomenon becomes prominent when TPS exists as a major component in the blends and thus causes better elongation at the break value, especially at 60/40 and 80/20 TPS/PS blends.

### 3.3. Fourier Transform Infrared Spectroscopy Analysis (FTIR)

**Figure 7:**
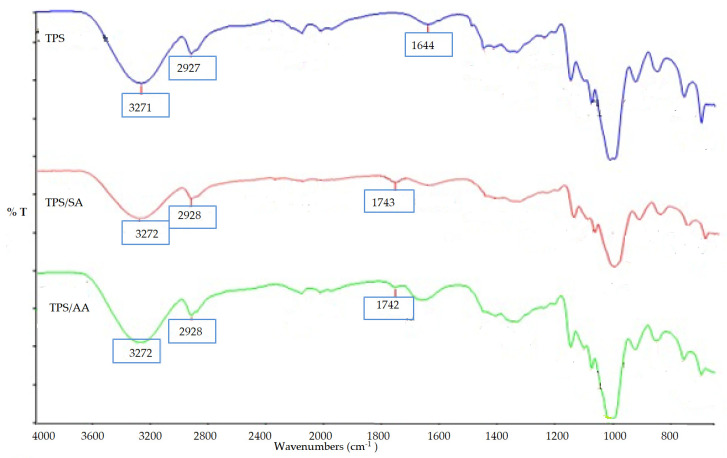
FTIR spectra of untreated TPS and TPS treated with SA and AA.

A FTIR test was carried out on three samples of TPS, untreated, treated with ascorbic acid, and treated with anhydride. Figure 7 shows that the band range from 3200 to 3700 cm^−1^ indicating the vibration stretching of hydrogen bonds between starch and glycerol hydroxyl groups. The strong peaks observed at 1000 cm^−1^ to 1100 cm^−1^ show the C–O bond stretching the C–OH group in the TPS. All three samples showed similar spectra except for both the SA and AA treatments, which corresponded to a new peak at 1743 cm^−1^ for SA treated and 1742 cm^−1^ AA treated. These new peaks show the formation of carboxyl and ester carbonyl bands for both treatments. The presence of a carbonyl peak in AA and SA confirmed that chemical linkages between ascorbic acid and acid anhydride and starch existed [31].

### 3.4. Morphological Study of PS/TPS Blends

**Figure 8:**
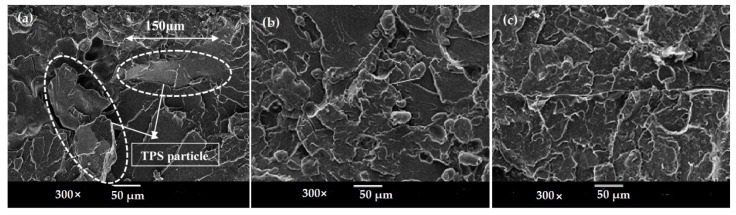
SEM micrographs of (**a**) untreated TPS/PS, (**b**) TPS/PS/SA, and (**c**) TPS/PS/AA with 20/80 proportion blends.

Morphology and the dispersion of polymers in the blends are the key factors that affect the properties of the blends. In general, the morphology of all the blends was observed at a magnification of 100× and 300× based on a 20/80 proportion of blend ratio. A scanning electron microscope (SEM) was used to observe the morphology behavior of the polymer blends and the dispersion in the blends system. Scanning electron micrographs of tensile fractured surfaces of untreated TPS/PS and treated TPS with SA and AA are shown in Figure 8. In Figure 8a, the SEM micrograph shows a clear separation at the interface between the TPS and PS component due to the incompatible blend between hydrophilic TPS and hydrophobic PS. TPS particles of approximately 150 µm showed poor and weak dispersion of TPS blends in Figure 8b. TPS/PS/SA showed good distribution of the blends with a smaller TPS particle size of 10 µm of in the PS component. The particle size was smaller due to well mixing, whereby TPS was able to flow, enabling the TPS to disperse and become embedded within the PS component. Good adhesion between phases allows TPS to exhibit a small globular shape in the PS component, which can be seen in Figure 8, leading to better compatibility. Treated TPS/PS with ascorbic acids in Figure 8 shows the improvement of surface and good distribution between the blends. The incorporation of ascorbic acid had enhanced the morphology of the blends system. The blend was well dispersed due to the enhanced plasticity of starch upon modification. These indicated better adhesion between the two materials. A previous study from Kahar et al. [32] reported that ascorbic acid and citric acid increase the plasticization and melt processing properties of TPS.

**Figure 9:**
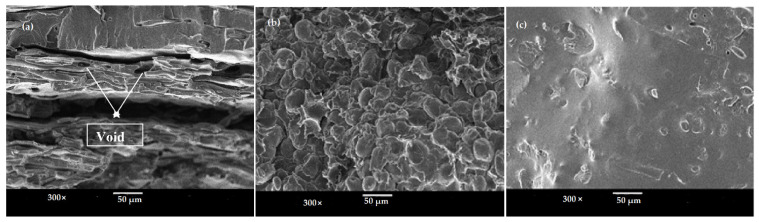
SEM micrographs of (**a**) untreated TPS/PS, (**b**)TPS/PS/SA, and (**c**) TPS/PS/AA with 80/20 proportion blends.

On the other hand, SEM micrograph for untreated and treated blends of 80/20 of TPS/PS blends ratio is shown in Figure 9. For untreated TPS/PS blends, higher TPS loading in the blending makes the surface rough and coarse, as seen in Figure 9a,b. This can be proved by some observable voids indicating poor interfacial interaction between the blends. The PS component does not tend to disperse homogeneously in the blends and leads to poor mechanical properties. Figure 9c indicates that the treated blend with higher loading TPS content improved the interfacial interaction and promoted good dispersion between the blends. Due to homogeneous dispersion, smaller particles show better distribution as compared to the untreated blends. The good interfacial adhesion between TPS and PS contributes to the breakdown of the PS component into the smaller sized dispersed phase. For 80/20 of TPS/PS blends, after being modified with ascorbic acid, they indicated that the TPS particles were mostly melted and fused together to form a continuous phase of plasticized TPS as shown in Figure 9c. The combined effect of glycerol as a plasticizer and modification with AA improved the plasticization of blends and exhibited finer particles with a good dispersion phase. PS are embedded well in the major TPS matrix and resulted in good compatibility. This result shows that the acidity of AA weakens the interaction between starch polymer chains onthe surface and thus improved the plasticization of TPS.

### 3.5. Thermal Analysis of PS/TPS Blends

**Figure 10:**
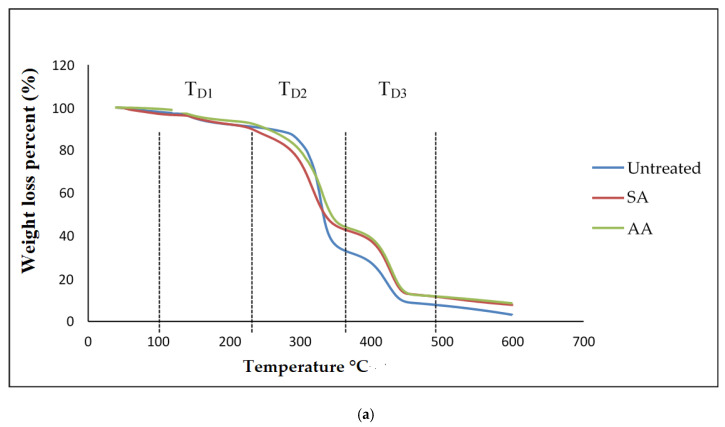

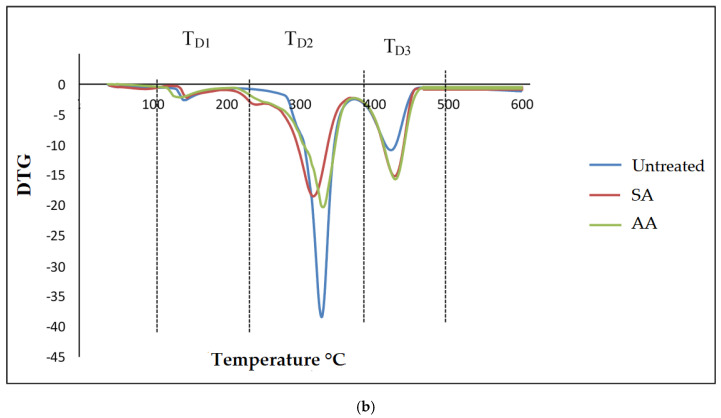
(**a**) TGA curves of PS/TPS blend with a ratio of 80/20 (TPS/PS). (**b**) DTG curves of blend with a ratio of 80/20 (TPS/PS).

**Figure 11:**
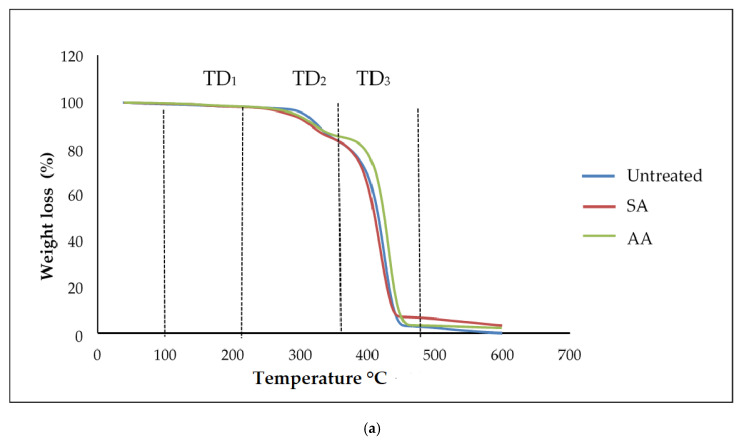

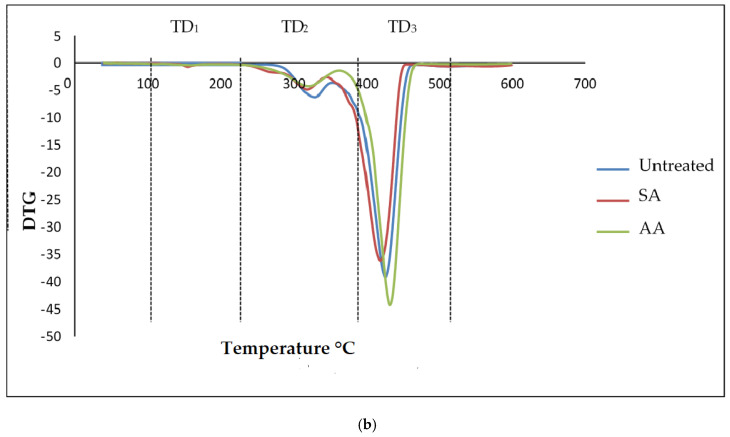
(**a**) TGA curves of PS/TPS blend with a ratio of 20/80 (TPS/PS). (**b**) DTG curves of blend with a ratio of 20/80 (TPS/PS).

Generally, thermogravimetric analysis (TGA) is a testing that is performed in order to determine the changes in weight loss with temperature. The TGA and DTG curves of untreated starch and starch treated with SA and AA, with ratios 80/20 and 20/80, are present in Figure 10 and Figure 11, respectively. There were three well-defined stages in the TGA curve summarizing the weight loss. The first stage occurred at 100–250 °C and indicates the evaporation of glycerol from the TPS/PS blends. Further, the weight loss gradually was increased as glycerol continued to evaporate from the TPS structure. The second stage occurred at 250–350 °C, and it can be attributed to the thermal degradation of starch. Similar results were observed during the characterization done by Mano et al. [33] in corn TPS/PE blends and cassava TPS/eggshell powder composite, respectively. Finally, the third stage was a result of PS degradation beginning at 350 °C. A further weight loss at a temperature above 450 °C can be described as thermal decomposition of PS, and a nearly complete decomposition was observed to occur at 500 °C. As seen in Table 1, the temperature at 5% weight loss was around 150–200 °C for higher TPS loading, which had lower thermal stability, indicating the evaporation of higher glycerol absorption in the TPS structure.

The slight shift in the temperature axis for untreated blends and treated blends was possibly caused by the modification on the starch prior to blending. TPS/PS treatment improved thermal stability of TPS by enhancing the adhesion between glycerol and starch. Hence, the heat was well distributed upon thermal introduction, and this was further improved with the presence of ascorbic acid and succinic anhydride in TPS/PS blends. However, increased TPS loading in AA treatment made the weight loss higher due to acidolysis reaction. Higher TPS loading treated with AA caused increased interfacial interaction between TPS particles and ascorbic acid and glycerol. On the other hand, an increasing amount of ascorbic acid caused acidolysis and the rigid structure of starch was lost completely [34]. Untreated blends have a lower degradation temperature, indicating that their thermal stability is less than treated blends. Low thermal stability of TPS and poor interaction at the interface between TPS and PS components might lead to this behavior. A similar result for the blends at 95% weight loss showed that untreated blends have a lower thermal stability compared to the treated blends.

### 3.6. Water Absorption Analysis

**Figure 12:**
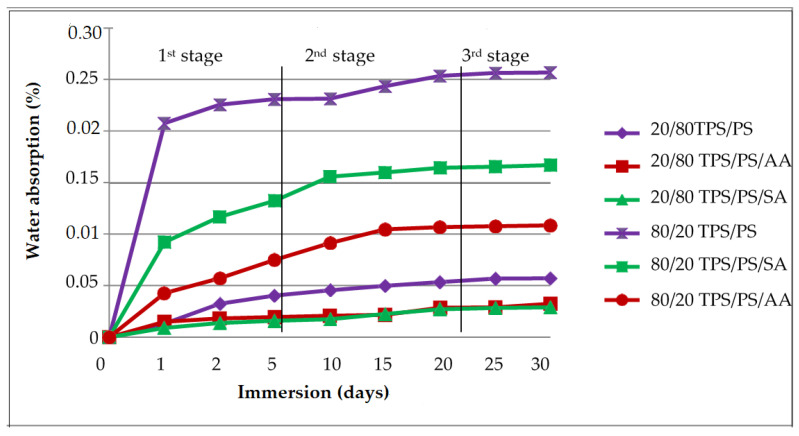
Water absorption of untreated and treated with AA and SA at two different ratios.

Figure 12 shows the water absorption effect of TPS/PS blends after immersion into water for different periods of time (1 month) for 80/20 and 20/80 TPS/PS blend ratios. The result was divided into three stages to examine the amount of water moisture that was absorbed by the blends. The first stage, indicating both the ratio for untreated and treated, shows the highest amount of water uptake due to the water molecule filling the space and void between the TPS-PS blends. Poor wetting between the hydrophilic nature of TPS and the hydrophobic PS phase generated more voids that allow water molecules to flow and store in the blend [35]. Water absorption of the blends increased with an increase in TPS loading. Furthermore, at high starch content, water could easily saturate the surface of the blend, penetrate into these voids, and be quickly absorbed by the starch, resulting in higher water absorption [36]. The second stage showed the slower penetration of water absorption as the reaction only occurs between the hydroxyl group of starch and water molecules. Another reason is that free hydrogen groups in TPS were available for this interaction, this leads to water uptake before it becomes saturated at the TPS surface. The last stage showed the slowest water uptake because of saturation of water absorption in the blend restricted the penetrating of water absorption in the blends.

When comparing the sample with and without treatment, blends treatment showed better water resistance compared to untreated blends. Treated blends with ascorbic acid (AA) formed an ester linkage between the hydroxyl group from the starch and ascorbic acids, thus increasing the hydrophobicity of the blends. Furthermore, it was reported that adding ascorbic acids yield a starch ester, which leads to crosslinking in the starch chain and results in reducing the void in the blends [37]. For TPS with SA treatment, it showed the slowest water uptake compared to the untreated blend. This is due to the ability of anhydride molecules to place themselves at the TPS-PS interphase, which was attributed to an increase in the interfacial adhesion strength. Hydroxyl groups of starch may react with anhydride functional groups and form ester linkages. These ester linkages compared to the hydroxyl group take significantly lower amounts of moisture.

## 4. Conclusions

In this study, TPS was blended with polystyrene with different blend proportions. Upon the blending, increasing TPS loading in the blends reduced the tensile strength and modulus of elasticity but increased the elongation at break value. Phase separation was clearly observed through the morphology studies especially at a high ratio of TPS loading. In addition, the thermal degradation temperatures were lower which reflects the lower thermal stability of TPS/PS blends.

However, modification of TPS/PS blends could have some effects on improving the properties of the TPS/PS blends via reducing the TPS hydrophilicity and enhancing the compatibility between TPS and PS phases. Treatment with succinic anhydride and ascorbic acid improved the tensile strength due to better interfacial adhesion between the TPS and PS components. Reaction between anhydride group with the hydroxyl group of TPS formed ester linkages and improve the surface adhesion between phases in the blends. The formation of ester linkages has been proved by inspection of the peak, which appears around 1743 cm^−1^ in FTIR spectra.

Acidity from ascorbic acids increased the plasticization of TPS and improved the diffusion of TPS in the polystyrene phase. Morphology of the treated TPS/PS blends showed improve in the dispersion of the TPS phase in the PS matrix. Furthermore, water uptake also decreased as the formation of ester linkages was hindering the possibility of free hydroxyl groups to bind with water molecules.

This work demonstrated that it is possible to use TPS as a partial replacement for conventional plastics. The modification with succinic anhydride and ascorbic acid were useful in improving the blends’ compatibility and processability without changing the fundamental properties of TPS. Further research on this present study will be focused on the effect of TPS on the biodegradability of the blends, as well as their potential impacts on the environment.

## Figures and Tables

**Table 1:** Composition of TPS blended with PS in different weight proportions.

	Samples TPS/PS	TPS (wt%)	PS (wt%)
Untreated Sago Starch	100/0	0	100
	80/20	80	20
	60/40	60	40
	40/60	40	60
	80/20	20	80
	0/100	0	100
Treated Sago Starch with Ascorbic acid	80/20	80	20
	60/40	60	40
	40/60	40	60
	20/80	20	80
Treated Sago Starch with Succinic Anhydride	80/20	80	20
	60/40	60	40
	40/60	40	60
	20/80	20	80

## Data Availability

This study did not report any data.

## References

[1] Vert M., Santos I.D., Ponsart S., Alauzet N., Morgat J.L., Coudane J., Garreau H. (2002). Degradable polymers in a living environment: Where do you end up?. Polym. Inter..

[2] Nair L.S., Laurencin C.T. (2007). Biodegradable polymers as biomaterials. Prog. Polym. Sci..

[3] Kahar A.W.M., Sarifuddin N., Ismail H. (2017). Structural, thermal and physico-chemical properties of high density polyethylene/natural rubber/modified cassava starch blends. Ira. Polym. J..

[4] Mohd Zain A.H., Kahar A.W.M., Noriman N.Z. (2016). Chemical-mechanical hydrolysis technique of modified thermoplastic starch for better mechanical performance. Proc. Chemist..

[5] Dos Santos R.D., Bardi M.A.G., Machado L.D.B., Dias D.B., de Silva L.G.A., Kodama Y. (2009). Influence of thermoplastic starch plasticized with biodiesel glycerol on thermal properties of PP blends. J. Ther. Anal. Calorim..

[6] Alias N.F., Ismail H., Kahar A.W.M. (2017). Properties of polyvinyl alcohol/palm kernel shell powder biocomposites and their hybrid composites with halloysite nanotubes. BioResources.

[7] Rodriguez-Gonzalez F.J., Ramsay B.A., Favis B.D. (2003). High performance LDPE/thermoplastic starch blends: A sustainable alternative to pure polyethylene. Polymer.

[8] Zheng Y., Yanful E.K., Bassi A.S. (2005). A review of plastic waste biodegradation. Cri. Rev. Biotechnol..

[9] Kumar M., Mohanty S., Nayak S.K., Parvaiz M.R. (2010). Effect of glycidyl methacrylate (GMA) on the thermal, mechanical and morphological property of biodegradable PLA/PBAT blend and its nanocomposites. Biores. Technol..

[10] Gaspar M., Benkő Z., Dogossy G., Reczey K., Czigany T. (2005). Reducing water absorption in compostable starch-based plastics. Polym. Degrad. Stabil..

[11] Ma X., Yu J. (2004). Studies on the properties of formamide plasticized- thermoplastic starch. Act. Polym. Sin..

[12] Huang M.F., Yu J.G., Ma X.F. (2004). Studies on the properties of montmorillonite-reinforced thermoplastic starch composites. Polymer.

[13] Mohd Zain A.H., Kahar A.W.M., Ismail H. (2019). Influence of electron beam radiation on structural and mechanical performance of thermoplastic cassava starch. Mater. Resear. Exp..

[14] Kahar A.W.M., Ismail H., Othman N. (2012). Effects of polyethylene-grafted maleic anhydride as a compatibilizer on the morphology and tensile properties of (thermoplastic tapioca starch)/(high-density polyethylene)/(natural rubber) blends. J. Vinyl Addi. Technol..

[15] Alias N., Ismail H., Wahab M.K., Ragunathan S., Ardhyananta H., Ting S. (2018). Physical, Tensile, and Biodegradability Properties of Cross-linked Polyvinyl Alcohol/Palm Kernel Shell Powder Biocomposites. BioResources.

[16] Bastioli C. (2001). Global status of the production of biobased packaging materials. Starch Stärke.

[17] Kahar A.W.M., Lingeswarran M., Amirah Hulwani M.Z. (2019). Plasticized jackfruit seed starch: A viable alternative for the partial replacement of petroleum-based polymer blends. Polym. Bull..

[18] Bhosale R., Singhal R. (2006). Process optimization for the synthesis of octenyl succinyl derivative of waxy corn and amaranth starches. Carbohyd. Polym..

[19] Bengtsson M., Koch K., Gatenholm P. (2003). Surface octanoylation of high-amylose potato starch films. Carbohyd. Polym..

[20] Reis K.C., Pereira J., Smith A.C., Carvalho C.W.P., Wellner N., Yakimets I. (2008). Characterization of polyhydroxybutyrate-hydroxyvalerate (PHB-HV)/maize starch blend films. J. Food Eng..

[21] Noorizzah I., Kahar A.W.M., Uylan D.N. (2016). Mechanical and Physical Properties of Polylactic Acid (PLA)/Thermoplastic Starch (TPS) Blends. J. Polym. Mater..

[22] Saw L.T., Rahim N.A.A., Viet C.X., Kahar A.W.M. (2015). Processing degradation of polypropylene-ethylene copolymer-kaolin composites by a twin-screw extruder. Polym. Degrad. Stabil..

[23] Russo M.A., O’Sullivan C., Rounsefell B., Halley P.J., Truss R., Clarke W.P. (2009). The anaerobic degradability of thermoplastic starch: Polyvinyl alcohol blends: Potential biodegradable food packaging materials. Biores. Technol..

[24] Singh J., Kaur L., McCarthy O.J. (2007). Factors influencing the physico-chemical, morphological, thermal and rheological properties of some chemically modified starches for food applications—A review. Food Hydrocoll..

[25] Mani R., Bhattacharya M. (2001). Properties of injection moulded blends of starch and modified biodegradable polyesters. Eur. Polym. J..

[26] Liu H., Xie F., Yu L., Chen L., Li L. (2009). Thermal processing of starch-based polymers. Prog. Polym. Sci..

[27] Taguet A., Huneault M.A., Favis B.D. (2009). Interface/morphology relationships in polymer blends with thermoplastic starch. Polymer.

[28] Godbole S., Gote S., Latkar M., Chakrabarti T. (2003). Preparation and characterization of biodegradable poly-3-hydroxybutyrate–starch blend films. Biores. Technol..

[29] Shogren R.L. (2003). Rapid preparation of starch esters by high temperature/pressure reaction. Carbohyd. Polym..

[30] Mohd Zain A.H., Kahar A.W.M., Hanafi I. (2018). Biodegradation Behaviour of Thermoplastic Starch: The Roles of Carboxylic Acids on Cassava Starch. J. Polym. Environ..

[31] Kahar A.W.M., Ismail H. (2016). High-density polyethylene/natural rubber blends filled with thermoplastic tapioca starch: Physical and isothermal crystallization kinetics study. J. Vinyl Add. Technol..

[32] Kahar A.W.M., Ismail H., Abdul Hamid A. (2016). The correlation between crosslink density and thermal properties of high-density polyethylene/natural rubber/thermoplastic tapioca starch blends prepared via dynamic vulcanisation approach. J. Therm. Anal. Calorim..

[33] Mano J.F., Koniarova D., Reis R.L. (2003). Thermal properties of thermoplastic starch/synthetic polymer blends with potential biomedical applicability. J. Mater. Sci. Mater. Med..

[34] Alessandra L., Da Róza M.D.Z., Antonio A.S.C., Antonio J.F.C. (2011). Thermoplastic starch modified during melt processing with organic acids: The effect of molar mass on thermal and mechanical properties. Ind. Crops Prod. J..

[35] Thomason J.L. (1995). The interface region in glass fibre-reinforced epoxy resin composites. Water absorption, voids and the interface. Compost.

[36] Ke T., Sun S.X., Seib P. (2003). Blending of Poly(lactic acid) and starches containing varying amylose content. J. App. Polym. Sci..

[37] Rahman M.R., Huque M.M., Islam M.N., Hasan M. (2008). Improvement of physico-mechanical properties of jute fiber reinforced polypropylene composites by post-treatment. Compos. Part A App. Sci. Manufac..

